# Remedy for Gleet

**Published:** 1833

**Authors:** 


### III. Remedy for Gleet.

Another correspondent, of the State of Pennsylvania, has sent us for insertion, the following recipe for this obstinate affection:

"There are few practitioners of much experience, who have not sometimes been perplexed by the obstinacy of this complaint. Numerous are the cases where, after inflammation, heat of urine, and every *painful* symptom have been easily subdued, and patient as well as physician have considered the chief difficulty overcome, there is nevertheless left behind a constant oozing of their glary fluid, which withstands every endeavor to stop it. The complainant becomes impatient and uneasy, whilst his attendant can only lament over the failure of his best exertions.

After repeated trial of the means commonly advised and adopted, and as often encountering only disappointment and chagrin, I was induced to prescribe opium conjoined with borax, in the following proportions: Opium grs. vi, sub. bor. sod.

3i, triturate and mix. Divide into xii powders. Take one every night and morning in thick syrup. The good effects from this treatment, will be very soon evident, and as far as my own experience goes, it is competent to remove any gleet whatsoever. If the powers of this recipe are fairly and generally tested, I am convinced it will supersede, as a general remedy, every medicine hitherto used."

